# Psychological and health behaviour outcomes following multi-gene panel testing for hereditary breast and ovarian cancer risk: a mini-review of the literature

**DOI:** 10.1186/s13053-022-00229-x

**Published:** 2022-06-22

**Authors:** Lindsay Carlsson, Emily Thain, Brittany Gillies, Kelly Metcalfe

**Affiliations:** 1 Drug Development Program, Princess Margaret Cancer Centre, 620 University Avenue, 8-132, Toronto, ON Canada; 2 Lawrence S. Bloomberg Faculty of Nursing, University of Toronto, Toronto, ON Canada; 3 Women’s College Research Institute , Toronto, ON Canada; 4 Bhalwani Familial Cancer Clinic, Princess Margaret Cancer Centre, Toronto, Canada; 5 Department of Molecular Genetics, University of Toronto, Toronto, Canada

**Keywords:** Genetic testing for cancer susceptibility, Hereditary breast and ovarian cancer, Panel testing, Psychological distress, Cancer screening and prevention, Breast cancer, Ovarian cancer

## Abstract

**Introduction:**

Knowledge of the genetic mechanisms driving hereditary breast and ovarian cancer (HBOC) has recently expanded due to advances in gene sequencing technologies. Genetic testing for HBOC risk now involves multi-gene panel testing, which includes well characterized high-penetrance genes (e.g. *BRCA1* and *BRCA2*), as well as moderate- and low-penetrance genes. Certain moderate and low penetrance genes are associated with limited data to inform cancer risk estimates and clinical management recommendations, which create new sources of genetic and clinical uncertainty for patients.

**Purpose:**

The aim of this review is to evaluate the psychological and health behaviour outcomes associated with multi-gene panel testing for HBOC risk. The search was developed in collaboration with an Information Specialist (Princess Margaret Cancer Centre) and conducted in the following databases: MEDLINE, EMBASE, EMCare, PsycINFO, Epub Ahead of Publication.

**Results:**

Similar to the *BRCA1/2* literature, individuals with a pathogenic variant (PV) reported higher levels of testing-related concerns and cancer-specific distress, as well as higher uptake of prophylactic surgery in both affected and unaffected individuals compared to those with variant of uncertain significance (VUS) or negative result. A single study demonstrated that individuals with a PV in a moderate penetrance gene reported higher rates of cancer worry, genetic testing concerns and cancer-related distress when compared to women with high penetrance PV. Analysis of cancer screening and prevention outcomes based upon gene penetrance were limited to two studies, with conflicting findings.

**Conclusion:**

The findings in this review emphasize the need for studies examining psychological and health behavior outcomes associated with panel testing to include between group differences based upon both variant pathogenicity and gene penetrance. Future studies evaluating the impact of gene penetrance on patient-reported and clinical outcomes will require large samples to be powered for these analyses given that a limited number of tested individuals are found to have a PV.

## Introduction

Knowledge of the genetic mechanisms driving hereditary breast and ovarian cancer (HBOC) has recently expanded due to advances in gene sequencing technologies. *BRCA1* and *BRCA2 (BRCA1/2*) account for only 20–30% of HBOC cases [1, 2], leading to the identification of other genes implicated in this hereditary syndrome. As a result, cancer genetic testing has shifted away from sequentially screening high-risk individuals for a limited number of well-characterized genes towards adopting multi-gene panel testing [3].

Currently, the clinical utility and validity of panel testing remains limited due to the minimal and often variable data informing age-specific risk estimates associated with several moderate-penetrance (MP) gene mutations [3–7]. There are few guidelines regarding the medical management of individuals harboring mutations in MP genes [3, 5, 8]. Panel testing also increases the likelihood of identifying variants of uncertain clinical significance (VUS), as well as variants where the clinical implications extend beyond the indication for testing. Multi-gene panel testing introduces new sources of clinical uncertainty, which may create challenging cancer screening and prevention decisions for patients and practitioners [3, 5, 9].

Initial studies examining patient outcomes with panel testing for hereditary cancer risk focused on exploring patient understanding and preferences [3]. These studies suggest that although patients are highly motivated to pursue testing, gaps in patient understanding exist and preferences surrounding return of results are variable [10–12]. These findings have raised concerns about the specific informational and support-based needs of individuals undergoing panel testing and identified challenges to traditional genetic counselling approaches [3, 13, 14].

Research examining the psychological impact and health behaviors of patients undergoing germline *BRCA1/2* testing described a cognitive process that follows result disclosure, where tested individuals must interpret their own cancer genetic risk in the context of their personal and/or familial cancer history [15]. The literature suggests that the type of genetic test result influences an individual’s risk perception [16–18]. Uncertain genetic risk associated with uninformative test results can negatively influence one’s cancer risk perceptions, psychological functioning, and uptake of cancer screening and prevention options [15, 19]. Extrapolating from the *BRCA1/2* literature highlights how the new sources of complexity and uncertainty associated with multi-gene panel testing may impact how patients respond to panel-based testing and underscores the need for research to be focused in this area. The aim of this review is to evaluate the psychological and health behaviour outcomes associated with multi-gene panel testing for HBOC risk.

## Methods

### Search strategy

The search strategy was developed in consultation with an Information Specialist at the University Health Network Library (Toronto, ON), who has expertise in conducting literature reviews, with a specialization in oncology. The following research questions guided this review: (i) How does multi-gene panel testing for HBOC risk impact the level of psychological distress of tested individuals? (ii) How does multi-gene panel testing for HBOC risk inform the cancer screening and prevention decisions of tested individuals? and (iii) Does the type of test result influence one’s psychological distress and/or uptake of cancer screening and prevention options? The search was conducted in November 2019 in the following databases: MEDLINE, EMBASE, EMCare, PsycINFO, Epub Ahead of Publication. An updated search was performed in March 2021. The following are examples of Medical Subject Headings (and associated keywords) used in this search: Neoplastic Syndromes, Hereditary/ (hereditary breast and ovarian cancer); Genetic Testing/ (multigene panel testing); Genetic Counseling/ (genetic screening OR genetic risk); Early Detection of Cancer/ ((prophylactic OR preventative) AND (mastectomy OR oophorectomy)); and Stress, Psychological/ (distress OR anxiety). Limitations were set in each database to ensure studies were published in the English language between January 2010 and March 2021 to align with the integration of panel testing into clinical care.

### Literature review and data extraction

**Fig. 1:**
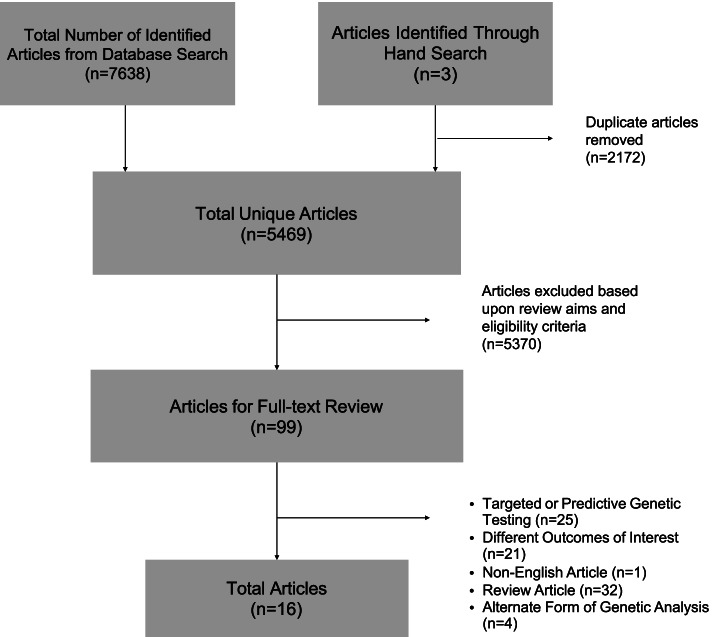
Literature search flowchart

A total of 5469 unique articles were identified through this search (Fig. 1). The titles and abstracts of these articles were manually screened by two independent reviewers (LC, BG, and ET), excluding studies that included any of the following criteria: (i) evaluating alternate forms of genetic testing and/or non-HBOC syndromes; (ii) evaluating clinical interventions and/or models of care; (iii) examining the familial implications of hereditary cancer genetic testing; (iv) pediatric and/or adolescent malignancies; (v) in-vitro and biomarker-focused studies; (vi) review articles or meta-analyses; (vii) clinical case reports, expert opinion articles, and/or clinical guidelines; and (viii) studies examining cancer screening modalities and surgical strategies in high-risk cohorts. A subset of 99 articles were then selected for full text review referencing the same exclusion criteria, from which a final set of 16 articles were selected for complete review and data extraction. The following data points were extracted: (i) study publication details; (ii) study design; (iii) sample description (including sample size); (iv) methods description (including survey tools and timing of assessments); (v) psychological outcomes and/or cancer screening and prevention outcomes (Tables 1 and 2 – condensed versions).

**Table 1:** Summary of psychological outcomes

Study	Country	Study design	Population	Select outcomes
**BRADBURY ET AL. (2016) **[20]	USA	Prospective study	Individuals > / = 18 years eligible for 25- gene panel testing, including *BRCA1/2* negative individuals (*n* = 28) and *BRCA1/2* untested (*n* = 21)	• No significant changes in general anxiety and depression [HADS], state anxiety [STAI], or breast cancer worry [IES] when compared pre- and post-disclosure
**ESTEBAN ET AL. (2017) **[21]	EU	Prospective longitudinal study	Individuals recruited from parent study FAMOSA completing multi-gene panel testing (25-genes) between Nov 2014 and Feb 2015. Cases had to fulfill either NCCN HBOC or Bethesda Lynch Syndrome criteria (*n* = 187)	• Cancer worry (CWS) did not change longitudinally (12-month follow-up) within subgroups (positive, negative and VUS genetic test result). Higher levels of worry were reported in the MP PV subgroup compared to those with a PV in a high risk gene at 1- week post-disclosure (*p* = 0.043); • Distress [MICRA Subscale] levels were significantly higher in the PV subgroup compared to the negative and VUS subgroups at 1 week, 3 months, and 12 months (*p* < 0.01). Higher levels of distress were reported in the MP PV subgroup compared to those with a PV in a high risk gene at 12-months (*p* = 0.026) • Uncertainty [MICRA subscale] did not change longitudinally (12- month follow-up) within subgroups (positive, negative and VUS genetic test result). Higher levels of uncertainty were reported in the MP PV subgroup compared to those with a PV in a high risk gene at 3- and 12-months post-disclosure (*p=0.038 and p* = 0.025) • Genetic testing-specific concerns [IES] did not change longitudinally (12-month follow-up) within subgroups (positive, negative, and VUS). The MP PV subgroup reported higher levels of concern than those with a PV in a high risk gene (*p* = 0.031)
**LUMISH ET AL. (2017) **[22]	USA	Cross- sectional study	Patients referred to a cancer genetics clinic due to HBOC risk and underwent genetic testing between June 2013 and May 2015. The study sample (*n* = 232) was divided into 6 subgroups based upon both their genetic test result (positive, negative, or VUS) and personal cancer history (affected or unaffected)	• Psychological impact of genetic testing [MICRA distress subscale] and distress [IES] was higher in the affected carriers compared to those with VUS or negative result (both affected and unaffected) (*p* < 0.05)
**IDOS ET AL. (2018) **[23]	USA	Prospective longitudinal study	Individuals from three genetics clinics who met clinical guidelines or risk estimate thresholds for genetic testing and underwent multi-gene panel testing (either 25-gene or 28-gene panel) (*n* = 2000)	• Distress and uncertainty [MICRA subscales] levels were significantly higher in the PV subgroup compared to the negative and VUS subgroups (*p* < 0.001)
**BRADBURY ET AL. (2020) **[24]	USA	Prospective longitudinal study	Individuals > 17 years old with prior *BRCA1/2 *negative testing and offered multi-gene panel testing between January 2014 and January 2015 (*n* = 249)	• General anxiety [HADS] and state anxiety [STAI] did not reveal longitudinal change within groups based upon genetic test result (positive, VUS or negative); • General depression [HADS] increased significantly from baseline to 12 months in the complete study sample (*p* < 0.001) and VUS (*p* < 0.01) subgroup; • Cancer specific distress [IES] increased significantly from baseline to 12 months in the complete study sample (*p* = 0.04) and in the VUS subgroup at 6 months (*p* = 0.04); • Uncertainty [MICRA] decreased significantly from baseline to 12 months in the entire study sample (*p* < 0.01) and negative (*p* < 0.001) and VUS (*p* < 0.01) subgroups at 6 months
**BREDART ET AL. (2019) **[25]	EU	Prospective longitudinal study	Individuals eligible for HBOC genetic testing recruited from genetics clinics in France, Germany, and Spain between November 2016 and April 2018 (*n* = 646)	• Specific psychosocial concerns [PAHC] were observed to decrease at 2 months post-disclosure compared to baseline in the complete study sample: 'hereditary predisposition' (*p* < 0.001), 'personal cancer' (*p* = 0.05), and 'children-related issues'(*p* < 0.001)

**Table 2:** Summary of behavioural outcomes

Study	Country	Study design	Population	Select outcomes
**ELSAYEGH ET AL. (2018) **[26]	USA	Retrospective cohort study	Individuals diagnosed with breast cancer who underwent multi-gene panel testing between 2014 and 2017 and received either a PV or VUS (*n* = 314)	• Higher rates of CPM in individuals with a *BRCA1/2* and non-*BRCA* PV compared to those with a VUS (*p* < 0.0001); • No significant difference in CPM rates between *BRCA1/2* carriers and non-*BRCA* carriers (*p* = 0.62)
**KURIAN ET AL. (2017) **[27]	USA	Cross-sectional study	Females (20–79) diagnosed with stage 0- II breast cancer between 2014 and 2015 who were reported to the undergone genetic testing (*n* = 666)	• PV were associated with higher rates of bilateral mastectomy compared to those with a negative result (OR 7.7; 95% CI, 3.9 to 15.3)
**LUMISH ET AL. (2017) **[22]	USA	Cross-sectional study	Patients referred to a cancer genetics clinic due to HBOC risk and underwent genetic testing between June 2013 and May 2015. The study sample (*n* = 232) was divided into 6 subgroups based upon both their genetic test result (positive, negative, or VUS) and personal cancer history (affected or unaffected)	• Amongst unaffected carriers (*n* = 14), 92.9% (*n* = 13) reported their results informed cancer screening and 7.1% (*n* = 1) informed prophylactic surgery decisions; • Amongst affected carriers (*n* = 11), 9.1% (*n* = 1) reported their results informed cancer screening and 18.2% (*n* = 2) informed prophylactic surgery decisions • Amongst unaffected individuals with a VUS (*n* = 20), 15% (*n* = 3) reported their results informed prophylactic surgery decisions and 35% (*n* = 7) reported their results informed cancer screening decisions • Amongst affected individuals with a VUS (*n* = 14), 7.1% (*n* = 1) reported their results informed prophylactic surgery decisions and 21.4% (*n* = 3) reported their results informed cancer screening decisions
**PEDERSON ET AL. (2018) **[28]	USA	Retrospective cohort study	Patients diagnosed with triple negative breast cancer (Stage I-IV) between September 2013 and December 2016 and underwent multi-gene panel testing (*n* = 226)	• Higher rates of CPM in the PV subgroup (88%) compared to the negative (20.1%) and VUS (21.4%) subgroups (*p* < 0.001). No significant difference noted between VUS and negative subgroups. *Note: the PV identified in this sample were limited to BRCA1/2 or PALB2*
**BUNNELL ET AL. (2017) **[29]	USA	Retrospective cohort study	Individuals who underwent multi-gene panel testing to evaluate HBOC risk between July 2013 and May 2014 (*n* = 136)	• Of the tested individuals, 8.8% (*n* = 12) received a genetic test result deemed medically actionable. The medical management recommendations changed for 10 (83.3%) of these individuals
**MURPHY ET AL. (2017) **[30]	USA	Retrospective cohort study	Women recently diagnosed with breast cancer and had genetic testing for hereditary cancer syndromes between January 2013 and August 2015 (*n* = 138)	• Amongst individuals with a VUS result, 29% (*n* = 2) had a bilateral mastectomy
**IDOS ET AL. (2018) **[23]	USA	Prospective longitudinal study	Individuals from three genetics clinics who met clinical guidelines or risk estimate thresholds for genetic testing and underwent multi-gene panel testing (either 25-gene or 28-gene panel) (*n* = 2000)	• Uptake of prophylactic surgery were reported to be higher in those with a PV (16%) compared to the negative and VUS subgroups (2.3% and 2.4%, respectively; *p* < 0.001)
**CHANG ET AL. (2019) **[31]	USA	Retrospective cohort study	Individuals deemed high risk for hereditary breast cancer (based upon NCCN guidelines or insurance criteria) and underwent genetic testing between January 2015 and August 2018 (*n* = 563)	• Higher rates of prophylactic oophorectomy were reported for individuals with a PV (9.8%) compared to those with a VUS (2.3%) or benign result (2.3%) (*p* = 0.03) • Higher rates of prophylactic oophorectomy were reported in individuals with a PV in a high risk gene (10.7%) compared to those in a moderate penetrance gene (1.7%) or genes deemed not-breast cancer specific (2.6%) (*p* = 0.05) • No significant differences were noted in reported rates of prophylactic mastectomy
**KURIAN ET AL. (2018) **[32]	USA	Retrospective cohort study	Females, aged 20–79 diagnosed with stage 0-II breast cancer (between 2013 and 2015) who were reported to have undergone genetic testing (*n* = 1316)	• The association between *BRCA1/2* variants and both surgeons' recommendation to undergo a prophylactic mastectomy and completing a prophylactic mastectomy was stronger than those with a non-*BRCA1/2* variant, VUS and negative test result (*p* < 0.001)
**FROST ET AL. (2018) **[33]	USA	Retrospective cohort review	Individuals who met NCCN guidelines for genetic evaluation for hereditary cancer between January 2015 and December 2016 (*n* = 671)	• The genetic test results for 23.3% (*n* = 156) of the study sample were associated with clinical management recommendations. Of these individuals, 39.7% (*n* = 62) received breast imaging recommendations; 36.5% (*n* = 57) individuals received ovarian cancer recommendations (TVUS ± CA125); and 37.2% (*n* = 58) individuals received prophylactic surgery recommendations
**VYSOTSKAIA ET AL. (2020) **[34]	USA	Retrospective cohort	Individuals who underwent genetic testing with a hereditary cancer panel (29-genes) between March 2016 and March 2018 were divided into two cohorts: (i) received a pathogenic or likely pathogenic test result (*n* = 161); (ii) did not receive a pathogenic or likely pathogenic result (control cohort) (*n* = 149)	• Amongst those with a PV in *PALB2, ATM, CHEK2, and/or NBN* and were recommended to undergo breast MRI (*n* = 66): 71% (*n* = 47) had completed an MRI; 26% (*n* = 17) reported future intention to have a breast MRI; • Amongst those with a PV in *BRIP1, RAD51C, and/or RAD51D* and were recommended to have a RRBSO (*n* = 9): 89% (*n* = 8) reported already undergone RRSO; 11% (*n* = 1) reported future intention to have an RRBSO
**CASKEY ET AL. (2020) **[35]	USA	Retrospective cohort review	Unaffected women at high risk of developing breast cancer and tested positive for a non-BRCA pathogenic mutation between February 2003 and March 2019 (*n* = 78)	• The genetic test results led to the following changes in clinical management: 3.8% (*n* = 3) initiated chemoprevention; 11.5% (*n* = 9) underwent prophylactic mastectomy; 88.5% (*n* = 69) initiated active surveillance
**BRADBURYET AL. (2020)** [24]	USA	Prospective longitudinal study	Individuals > 17 years old with prior BRCA1/2 negative testing and offered multi-gene panel testing between January 2014 and January 2015 (*n*=249)	• Future intention to undergo mammography declined significantly from baseline to 12 months in the VUS (*p* < 0.01) and negative result (*p*=0.04) subgroups. • Future intention to undergo breast MRI declined significantly from baseline to 12 months in the negative result subgroup (*p*=0.02).

### Quality assessment

The Standard Quality Assessment Criteria for Evaluating Primary Research Papers from a Variety of Fields developed by Kmet and colleagues (2004) was used to assess the quality of articles [36]. This assessment involved an evaluation of the key aspects of the research design, analysis, and reported results for each of the final articles. Quality assessments were completed by two reviewers for each of the final 16 articles. Discordant quality assessments were resolved by a third reviewer conducting a quality assessment.

## Results

Sixteen research studies met the eligibility criteria, including 5 prospective, 2 cross-sectional, and 9 retrospective cohort studies (Tables 1 and 2). Methodological differences between studies limited the findings of this review to a descriptive analysis. Specifically, inconsistent reporting of key participant and clinical details (familial and clinical variables, and genetic testing details) and variability in study methods and the clinical populations. All articles met quality review assessment thresholds by two independent reviewers [36]. The median quality assessment for the final 16 articles was 0.90 (range = 0.70–1.0).

Sample sizes ranged between 49 and 2000, with a total of 7781 participants enrolled across the 16 studies. Participants were predominantly female (93%) and Caucasian (61%). The mean age of participants ranged between 47.7 to 55.3 years. Amongst the 8 studies that reported education levels of study participants [20, 22–27, 32], the majority of study participants (63%) were college or university educated. Eight studies exclusively evaluated panel testing [21, 23, 24, 26, 29, 34], the remaining studies included participants who underwent either targeted (e.g. *BRCA1/2*) or panel testing. Five studies included individuals diagnosed with breast cancer [26–28, 30, 32] and eleven studies included affected and unaffected individuals [20–25, 29, 31, 33–35]. The majority of studies included in this review were conducted in the United States (*n* = 14).

### Psychological outcomes (Table 1)

Four prospective studies evaluated changes in psychological outcomes following multi-gene panel testing [20, 21, 24, 25], and two studies described psychological outcomes of affected and unaffected individuals from clinical cancer genetics programs at a single time point post-disclosure [22, 23]. Psychological outcomes included: anxiety and depression (State Trait Anxiety Inventory [STAI] [20, 24] and Hospital and Depression Scale [HADS] [20, 24]), cancer worry (Cancer Worry Scale [CWS]), cancer-related distress (Impact of Event Scale [IES] [21, 22, 24]), and genetic testing specific concerns (Multidimensional Impact of Cancer Risk Assessment [MICRA] [21–24] and Psychosocial Aspects of Hereditary Cancer [PHAC] [25]). Prospective studies included pre- and post-test evaluation [20, 25] and longitudinal follow-up (1-week to 12-months post-disclosure) [21, 24]. Study participants were recruited from cancer genetics programs, only three study explicitly stated that participants met NCCN guidelines for HBOC testing [21, 28, 33].

#### Anxiety and depression

Two studies measured changes in anxiety and depression levels over time among all tested individuals in the study sample and between subgroups based upon test result (pathogenic variant (PV), VUS or negative result) [20, 24]. No significant changes in state and general anxiety or general depression were observed post-disclosure compared to baseline levels [20]. In their follow-up study, Bradbury and colleagues [24] observed a significant increase in depression levels amongst all study participants at 12-months post-disclosure. Although the change in depression levels was statistically significant (*p* < 0.01) at 12-months it did not reach clinical significance (HADS score of < 8/21). No between group differences based upon variant pathogenicity were observed in anxiety and depression levels across 12-month follow-up [24].

#### Cancer worry

A single study used the CWS to evaluate cancer worry longitudinally [21]. No significant changes in cancer worry were noted over 12-month follow-up and there were no between group differences observed based upon genetic test results (positive, negative, VUS). Levels of cancer worry were higher at all post-disclosure time points in the moderate penetrance variant subgroup compared to high penetrance subgroup, however, was only significant at 1-week post-disclosure (*p* = 0.043) [21].

#### Cancer related distress

Three studies used the IES tool to measure changes in cancer-related distress levels over time [20, 21, 24]. Pre- and post-disclosure comparison did not reveal significant changes in cancer-related distress [20]. No differences in cancer-related distress were observed longitudinally (3 time points) between groups classified by test result (positive, VUS, and negative), but the moderate penetrance variant subgroup was shown to have higher mean IES scores compared to the high penetrance subgroup at 12 months post-disclosure (30.67 vs 10.71, *p* = 0.031) [21]. Bradbury and colleagues [24] focused on within group change over 12-month follow-up and observed no changes in cancer-related distress levels in the PV and negative test result subgroups. However, VUS carriers reported significantly elevated cancer-related distress levels at 6-months post-disclosure compared to baseline (*p* = 0.04).

One cross-sectional study found that PVs were associated with higher mean scores on the IES tool when measured 13 months post-disclosure compared to VUS and negative result subgroups [22]. Lumish and colleagues [22] considered the interaction between personal cancer history and genetic test result in their analysis. Unaffected carriers with a PV reported statistically elevated levels of distress compared to affected individuals with a PV, as well as when compared to unaffected and affected individuals with a VUS or negative result (*p* < 0.05).

#### Genetic testing specific concerns

Two studies [20, 21] used the MICRA tool to measure changes in genetic testing specific concerns over time among all tested individuals in the study sample and between subgroups based upon both the test result (pathogenic, VUS or negative result) and gene penetrance. Pre- and post-disclosure evaluation did not reveal significant changes in testing concerns [20]. Between group differences in testing-specific distress (MICRA subscale) was observed longitudinally when subgroups were classified by type of test result and gene penetrance [21]. Higher levels of distress were associated with a PV compared to VUS and negative test result subgroups (*p* < 0.01) at all time points (1-week; 3- and 12-month post-disclosure). Moderate penetrance PV were also observed to be associated with higher reported levels of distress compared to those with a high penetrance PV at the 12-month time point (17.67 vs 6.59, *p* = 0.026).

Two studies evaluated between group differences on the MICRA tool based upon variant pathogenicity at a single follow-up time point [22, 23]. Idos and colleagues [23] found that a PV was associated with higher reported levels of testing concerns compared to negative and VUS subgroups. The VUS subgroup was observed to have higher levels of testing-related uncertainty (MICRA subscale) compared to the negative result subgroup (*p* = 0.017). Similarly, Lumish et al. [22] observed higher genetic testing specific distress (MICRA distress subscale) in the unaffected carrier subgroup compared to affected carriers, as well as when compared to affected and unaffected individuals with VUS or a negative test result (*p* < 0.05).

One study prospectively evaluated changes in PAHC scores over time (pre- and 2 months post-disclosure) among all tested individuals and between group differences based upon gene panel result (pathogenic *BRCA1/2* variant; non-*BRCA1/2* pathogenic variant; VUS; and negative result) [25]. Overall, concerns related to ‘hereditary risk’ (*p* < 0.001), ‘personal cancer risk’ (*p* < 0.05), and ‘children-related considerations’ (*p* < 0.001) decreased over time in the study sample. Between group differences based upon the genetic test result were observed pre- and 2-months post-disclosure in concerns related to ‘hereditary risk’ and ‘familial and social issues’.

### Behavioral Outcomes (Table 2)

Five studies examined uptake of prophylactic surgery in patients diagnosed with breast cancer [26–28, 30, 32], and five studies evaluated the medical recommendations and patient-reported uptake of prophylactic surgery of patients reviewed in clinical genetics programs (affected and unaffected individuals) [23, 29, 31, 33, 35]. Three studies evaluated cancer screening uptake after result disclosure in patients of a cancer genetics clinic, including both affected and unaffected individuals [22, 24, 34].

#### Prophylactic surgery: breast cancer patient cohorts

Across studies, rates of contralateral prophylactic mastectomy (CPM) in women with breast cancer was higher in individuals with a PV compared to VUS and negative test result subgroups [26–28, 32]. Kurian et al. [27] found that uptake of CPM is more likely in individuals with a PV compared to a negative result (OR, 7.7; 95% CI, 3.9 to 15.3). In their 2018 study, Kurian and colleagues [32] expanded their analysis to include a subgroup of individuals with PV in genes other than *BRCA1/2*. Similar to their findings in 2017, a *BRCA1/2* PV was more strongly associated with both patient consideration of a CPM (*p* < 0.001), as well as a surgeon’s management recommendation for a CPM (*p* <  = 0.001) when compared to the remaining three categories (PV in a non-*BRCA* gene, VUS, and negative result). This contrasts findings from Elsayegh and colleagues [26] who reported that carriers of a PV (*BRCA1/2* and non-*BRCA1/2*) were more likely than VUS carriers to undergo a CPM (*p* < 0.0001), with no statistical difference found between *BRCA1/2* and non-*BRCA1/2* PVs. Observed rates of CPM in the VUS and negative subgroup ranged between 21.4%-30.2% and 20.1%-35.3%, respectively [28, 30, 32].

#### Prophylactic surgery: clinical genetics patient cohorts (unaffected and affected)

Overall, higher rates of prophylactic mastectomy and oophorectomy were associated with a PV compared to those with a VUS or negative test result [23, 31]. This association was found to be statistically significant at 13 months (*p* < 0.001) and 27.1 months (*p* = 0.03) post-disclosure in two studies [23, 31]. Chang and colleagues [31] found prophylactic oophorectomy rates were higher with PV in high penetrance genes (10.7%) compared to moderate penetrance (1.7%) and low risk (2.6%) genes (*p* = 0.05). This association was not observed in rates of prophylactic mastectomy. Two studies reported rates of prophylactic surgery in a cohort of individuals with non-*BRCA* PV [34, 35], supporting patient adherence to medical recommendations following result disclosure.

Two additional studies evaluated changes in clinical management plans following multi-gene panel testing. Frost and colleagues [33] observed a change to the clinical management plan in 23.3% of patients who underwent genetic testing. This included prophylactic surgery in 37.1% of patients and chemoprevention in 0.1% of patients. Conversely, Bunnell et al. [29] identified an actionable variant in only 8.8% (*n* = 12) of their sample, and the recommendations for clinical management (screening and/or prophylactic surgery) changed for 10 of those individuals. Of interest, 8 of the 12 individuals had non-*BRCA* PVs which informed medical recommendations.

#### Cancer screening

Three studies evaluated patient reported uptake and/or intention regarding cancer screening and prevention options in a cohort of affected and unaffected individuals referred to a cancer genetics clinic [22, 24, 34]. Lumish and colleagues [22] observed that a higher proportion of unaffected individuals with a pathogenic variant (92.9% vs 9.1%) or VUS (35% vs. 21.4%) reported that their test result impacted their cancer screening activities compared to affected individuals with a PV or VUS. Caution is warranted given the small number of individuals in each group. Bradbury and colleagues [24] similarly evaluated patient intention toward cancer screening and prevention but looked at within group changes over a 12-month follow-up (baseline, 1 week, 6- and 12-months post-disclosure). Individuals with a VUS and negative panel test result reported a significantly reduced intention to undergo breast cancer screening at 12 months. This trend over time was not observed in individuals with a PV. Finally, Vysotskaia et al. [34] evaluated changes in medical recommendations following a genetic test result, as well as patient adherence to those recommendations. Recommendations for screening MRI increased (42% to 82% post-disclosure) in individuals with a PV in *PALB2*, *ATM*, *CHEK2*, and/or *NBN*, which was associated with high rates of compliance (97%) [34].

## Discussion

The findings in this review emphasize the need for studies examining psychological and health behavior outcomes associated with panel testing to include between group differences based upon both variant pathogenicity and gene penetrance. Analyzing findings with both classification approaches will be critical in understanding how the clinical uncertainty associated with specific types of results impact psychological, screening and prevention outcomes in this population. Similar to the *BRCA1/2* literature, individuals with a PV reported higher levels of testing-related concerns and cancer-specific distress, as well as higher uptake of prophylactic surgery in both affected and unaffected individuals compared to those with VUS or negative result. Interestingly, individuals with MP PVs had higher rates of cancer worry, genetic testing concerns and cancer related distress when compared to women with high penetrance PV [21, 23]. Analysis of health behavior outcomes based upon gene penetrance was limited, with conflicting findings [26, 31, 32], and thus further research is needed.

Future studies designed to evaluate between group differences based upon gene penetrance will require large sample sizes to power such analyses given that the observed frequency of PVs is low. Depending on the clinical population and panel test used, PVs are observed in only 7–12% of tested individuals, which limits the ability to look at differences based upon penetrance due to the small numbers in each subgroup [23, 24, 37, 38]. In this review, the number of individuals carrying a MP PV was variable across study samples, ranging from 4 *ATM* carriers (11% of pathogenic variants) [21] to 12 MP (*ATM, CDH1, CHEK2, PALB2, NBN, NF1,* and *STK11*) carriers (17% of pathogenic variants) [23]. In addition, classification of MP variants was not consistent across studies. For example, *PALB2* was classified as both a MP and high penetrance variant in different studies [21, 31]. Thus, caution is warranted when interpreting these findings given the small number of individuals in each subgroup and the lack of uniform classification of variants.

Despite the low expected frequency of PVs with panel testing, initial work by Kurian and colleagues [38] in a *BRCA1/2* negative cohort, found that of the 15 PVs identified, 14 were actionable and informed medical management. This aligns with the observed changes in clinical management of individuals found to have a PV in this review [29, 33]. In both studies [29, 33] challenges were reported when formulating management plans for MP PVs and newly identified genes (e.g. *BARD1* and *NBN*) due to a lack of available medical management guidelines [29, 33]. This supports the need for individualized genetic counselling, as clinical expertise is required to provide recommendations in the absence of guidelines, by drawing on relevant literature in the context of the patient’s personal and familial history. Given limited genetic counseling resources and an increasing proportion of panel-based testing offered by non-genetics healthcare providers, post-test counseling with a certified genetic counselor may provide the optimal setting for this discussion [39].

Results from this review indicate that while more individuals with a PV undergo preventative surgery following panel testing compared to those with a VUS or negative result, a VUS result may inform cancer screening and prevention decisions. Kurian and colleagues [32] found rates of prophylactic surgery to be approximately 30% in affected patients with VUS and negative test results. These authors argued that the similar rates of prophylactic surgery observed between VUS and negative cohorts was suggestive that those with a VUS were not overestimating their cancer risk. These findings may support this claim, but it is worth considering that 30.2% of participants with a VUS opted to have prophylactic surgery, when it was recommended by the surgeon in 14.1% of cases [32]. Across studies in this review, VUS results were found to inform cancer screening and prevention decisions in approximately one-third of unaffected patients [22, 28, 30, 32]. As such, further work is needed to explore patient interpretation of cancer risk after receiving a VUS result and the perceived utility of these variants. Currently, there is limited data regarding other factors that may contribute to a patient’s risk management decision, such as current disease attributes, family history of cancer, as well as cancer and genetic testing related distress. Understanding patient decision-making surrounding cancer screening and prevention is further complicated by the inconsistent reporting of gene variants. Lumish and colleagues [22] noted that 62.5% of VUS were in a high penetrance gene, which may have contributed to the perceived utility of these variants. Consistent reporting of the gene where the VUS was identified may illuminate differences in health behavior outcomes and also reinforce the need for patient follow-up for variant reclassification updates.

Finally, screening for heightened levels of distress requires measuring these outcomes with tools that include clinical thresholds, such that clinically significant distress can be identified and appropriately managed. The *BRCA1/2* literature suggests that the majority of patients do not experience persistent psychological distress but estimates that 20–25% of tested individuals report long-term negative affective outcomes [15, 19]. Thus, as we evaluate psychological outcomes in the context of panel testing, it is important to question whether these observed differences in psychological outcomes actually translate into clinically meaningful differences. Lumish and colleagues [22] found the mean IES score of the unaffected carrier subgroup was significantly higher than all remaining subgroups 13 months post-disclosure (*p* = 0.01), yet the level of distress was considered ‘mild’ (< 25) on the IES tool, and not clinically significant. This can be contrasted with the findings from Esteban et al. [21], where the MP subgroup reported a mean score on the IES-R tool at a level where symptoms of distress may be present, suggesting clinical significance. Thus, it is important that when between group differences are evaluated for statistical differences, that they are also evaluated for clinical significance. Identifying subgroups at increased risk of experiencing clinically meaningful distress may assist in providing tailored genetic counselling and identifying supportive resources for those individuals that are at higher risk of experiencing negative psychological outcomes.

### Limitations

This literature review has several limitations. First, due to variable reporting of participant details and methodological differences, this review was limited to a descriptive analysis. Although, the authors followed a rigorous and systematic approach when conducting this literature search and synthesis of findings, this review was not pre-registered for PROSPERO. Second, this review was limited to publications in the English language, with most of the articles published by research teams in the USA (*n* = 14), which potential limits scope and relevance of the findings. In addition, across the studies in this review, the majority of participants were Caucasian women, who were highly educated, which may limit the generalizability of the findings. Finally, the conclusions drawn in this review are based upon the classification of variant pathogenicity and gene penetrance published in the included articles, which reflects the knowledge and guidelines at the time of each publication.

## Conclusions

The findings in this review highlight the importance of considering both variant pathogenicity and gene penetrance when exploring the impact of panel testing on psychological and health behavior outcomes. With the growing trend towards multi-gene panel testing, healthcare providers should be cognizant of individuals who are at risk for increased cancer worry and distress, as well as those who may overestimate their cancer risk and undergo inappropriate risk reduction surgery. Further research is needed to explore the factors that contribute to heightened levels of cancer worry and distress, the different personal demographics and clinical variables that inform cancer screening and prevention decisions, and the impact of supportive resources and counselling.

## Data Availability

Not applicable.
